# A New NVM Device Driver for IoT Time Series Database

**DOI:** 10.3390/mi13030385

**Published:** 2022-02-27

**Authors:** Tao Cai, Yueming Ma, Peiyao Liu, Dejiao Niu, Lei Li

**Affiliations:** School of Computer Science and Communication Engineering, Jiangsu University, Zhenjiang 212000, China; pyaoliu@163.com (P.L.); lilei@ujs.edu.cn (L.L.)

**Keywords:** NVM storage system, storage device driver, IoT, IoT time series database, I/O software stack

## Abstract

Numerous IoT devices in IoT systems collect data concurrently, which brings great challenges to IoT time series databases to store and manage these data. NVM device has high read–write speed compared with HDD and Flash-based SSD, and it is a possible way to solve the storage bottleneck. However, there are some limitations that should be solved such as the overhead of the I/O software stack for NVM devices and the lack of optimization for IoT time series databases in a Linux environment. By analyzing the characteristics of IoT time series databases and NVM devices, we optimized the device driver of NVM in Linux and provide a new structure of a NVM device driver for IoT time series databases. A multi-queue management strategy and a lightweight load balance mechanism based on frequency were designed to improve the concurrency and efficiency of NVM device drivers. The prototype of an IoT-oriented NVM device driver named TS-PMEM was implemented based on an open-source NVM device driver. Six prototypes were used for evaluation with YCSB-TS, a test tool for time series databases. Results showed that TS-PMEM can improve write throughput of the time series databases by 18.6%, query throughput by 10.6%, and reduce the write latency by 8.3% and query latency by 6.4%.

## 1. Introduction

There are many IoT devices in IoT systems, and they currently collect a large amount of time series data. How to store and manage them effectively is an important problem for IoT systems. In general, powerful servers and the IoT time series databases [1] for storing time series data generated by IoT devices are used to solve this problem. However, there are still great challenges in how to store and process the large amounts of time series data concurrently and quickly [2]. The HDD has slow read and write speed, which makes it difficult to support high-speed and concurrent storage and management of massive time series data in IoT systems. The Flash-based SSD also has several drawbacks such as the low write endurance and the write amplification. Next, the IoT time series database faces serious storage wall problems. Non-volatile memory (NVM) is a new type of storage device such as PCM (phase change memory) [3], STT-RAM (shared transistor technology random access memory) [4], the latest technology Intel 3D-XPoint [5], and the commercial Intel Optane DC persistent memory [6], etc. They have the advantages of byte addressable, long lifetime, low dynamic energy consumption, and close to DRAM (dynamic random-access memory) read–write speed, which is a good choice to improve the efficiency of the IoT time series database. However, high read–write speed of NVM also brings great challenges to the I/O stack of computer systems. There are many studies that have shown that the current I/O system software stack for NVM devices will cause 94% latency such as file systems, generic block layers, device drivers, etc. [7]. In addition, unlike NVMe drivers for the Flash-based SSD, the driver of newly commercial Intel Optane DC persistent memory still uses a single-queue to process I/O requests, which causes a performance bottleneck for IoT time series databases that need to store and process large amounts of IoT time series data. Therefore, it is necessary to improve the efficiency of the current NVM device driver for IoT time series databases.

Unlike social networks, scientific experiments, and log systems, there are many IoT devices in IoT systems and they concurrently and regularly collect data in a standardized form. The IoT time series database is a popular software to store and manage these data. In general, IoT time series databases face several challenges.

(1) Write operations occur far more than read operations. A large number of IoT devices continuously collect data in a standardized form and store them into IoT time series databases, so the write frequency is very high. Meanwhile, read operations of IoT time series databases only occur in query operations initiated by the users randomly or regularly, and its frequency is much less than write operations. Then, the IoT time series database needs to ensure write operations occur prior to read operations and can continuously store a large number of data.

(2) The data amount of a single write operation is small and regular. Every IoT device in the IoT system collects and stores data at a fixed frequency, which makes the write operation obviously regular and requires a stable I/O performance of the IoT time series databases. At the same time, the amount of data collected by a single IoT device is generally small each time, usually including a timestamp and a value, which requires the storage system to have a high performance to write and manage small data.

(3) The acquisition frequencies of different IoT devices are different. Each IoT device has a certain acquisition frequency, and the I/O software stack of IoT time series databases can be optimized by this characteristic.

(4) The time range query is very popular in IoT time series databases. Then, the read operation is very simple and the read performance of the IoT time series databases should be optimized for this kind of query.

(5) There is no update operation and single data deletion operation in IoT time series databases. IoT systems generally require the preservation of all IoT time series data within a long-time range. Once a single data is written, it cannot be modified or deleted; only old data can be deleted in batch after a fixed time interval.

These features should simplify the management of the IoT time series database and can be used to improve the efficiency of the IoT time series database. At the same time, although NVM devices have advantages compared with HDD and Flash-based SSD, their read and write speed are still lower than DRAM. Furthermore, there is still a gap of concurrency between NVM devices and DRAM due to the limitations of the current I/O system software stack and others. Then, it is not a good choice to replace Flash-based SSD or HDD with NVM devices directly to take read and write speed advantages of NVM devices. The device driver is the lowest level in the I/O system software stack of NVM devices and has a great impact on the performance of the IoT time series database based on NVM devices. Therefore, it is necessary to study a new NVM device driver according to the characteristics of IoT time series databases and NVM devices.

This paper makes the following contributions:

1. Given the large concurrent write operations in IoT time series databases, several queues are used in NVM device drivers to manage I/O requests, respectively. A new I/O request structure named Tio was designed to contain the characteristics of the IoT time series data, and multiple threads were used to manage the Tio in parallel. This can effectively improve the concurrency of the NVM device driver and take advantage of the read and write speed of the NVM device.
2. According to the different acquisition frequencies of IoT devices, a lightweight load balance mechanism based on frequency was designed. This can improve the efficiency of I/O management in NVM device drivers and increase the throughput rate and reduce the latency of IoT time series databases.
3. A prototype of new NVM device driver for IoT time series databases named TS-PMEM was implemented. Two popular time series databases and one IoT time series database based on NVM devices from our group [8] were used for testing by using a general test tool for time series databases named YCSB-TS, and compared with the popular NVM device driver named PMEM from Intel. The results showed that TS-PMEM can increase the write throughput of time series databases by 18.6%, improve the query throughput by 10.6%, reduce the write latency by 8.3%, and decrease the query latency by 6.4% in the maximum compared with the current NVM device driver.

## 2. Related Works

### 2.1. NVM Simulator and Driver

Simulation is the popular approach in NVM research when there is no commercial NVM device. Meanwhile, it can also be used to implement the device driver for NVM devices. VSSIM is a Flash-based SDD simulator based on virtual machine, which tracks the calibration and precision replay and evaluates the performance of the actual system [9]. VSSIM can flexibly model channel number, channel amount, block size, page size and the plane of each chip, programming, erasure, read delay, and channel switching delay. It also allows users to measure the performance of the host and Flash-based SSD under various design options. MQSim is a new multi-channel Flash-based SSD simulator [10] that can accurately simulate the I/O speed of a Flash-based SSD based on the SATA interface and high bandwidth protocols. It can also simulate the I/O latency of Flash-based SSD. SimpleSSD is a high-fidelity simulation software [11] that can simulate software and hardware characteristics more lifelike than the traditional Flash-based SSD simulator. At the same time, the non-descriptive characteristics of storage internals are simplified. Compared with the existing Flash-based SSD simulators, it can be easily integrated into the publicly-available computer system simulators and evaluate the performance with a variety of storage technologies and microstructures. Amber is a new simulation framework based on SimpleSSD [12]. Through detailed modeling of the whole system, the system is simulated accurately. It simulates various flash memory technologies in embedded CPU kernel, DRAM, and Flash-based SSD, and runs in a computer system simulator by supporting data transmission simulation. CPS-SIM is a Flash-based SSD simulator with an accurate clock that simulates the interior of Flash-based SSD and reports on its time and usage to find the best hardware configuration in Flash-based SSD [13]. By supporting concurrency, CPS-SIM provides a flexible environment for Flash-based SSD firmware. It can drive multiple flash memory chips and arrange data transmission through multiple buses. The DDR2 controller was modified to simulate NVM to implement a NVM device based on DRAM named Moneta [14]. The internal parameters of NVM devices can be simulated accurately. In order to reduce the impact of software overhead on computer performance and improve the efficiency of computer systems, the interface between computers and NVM device simulators has also been modified. However, due to the lack of commercial NVM chips, the accessibility of Moneta is limited. Quartz is a lightweight NVM device simulator that focuses on the I/O performance simulation [15]. While it effectively simulates the latency and bandwidth characteristics of various NVMs and evaluates their performance and its impact on application performance, it does not focus on low-level hardware details such as specific NVM devices, embedded processors, and memory, but only on modeling their key performance characteristics. OpenExpress is a NVM device simulator based on DRAM [16]. The internal structure of NVM devices is simulated by using multiple DRAM chips and multiple I/O channels, which supports scalable I/O commits, and the management list of many NVMe commands is realized. NVMain is an architecture-level main memory simulator [17] that can also simulate NVM devices and integrate them into the entire computer system simulator. Because it is an architecture level NVM device simulator, it is capable of accurately simulating the time and energy consumption of NVM memory devices. NVMain 2.0 is an improvement on NVMain [18], which has a high degree of flexibility and a user-friendly interface. Compared with the existing NVM device simulators, NVMain 2.0 has many new features such as the fine-grained management model and the MLC support. It has a convincing simulation for the read–write speed of NVM, and could support sub-array-level parallelism and fine-grained refresh. DRAMsim is a highly configurable NVM device simulator that can be integrated into a variety of computer system simulators or as a standalone simulator [19]. In addition, DRAMsim 2 [20] and Zsim [21] are nuclear NVM device simulators that use DRAM to simulate NVM. Ramdisk is the Linux kernel’s tool for partitioning DRAM to simulate NVM, improving the input and output performance of storage systems. PMEM is the open-source NVM device driver from Intel for Optane DC and it can also use the DRAM to simulate the NVM device without Optane DC. However, it still uses a single queue to manage I/O requests, which will affect the efficiency of the NVM device driver [22].

Today, most NVM device simulators or drivers still face an efficiency limitation with a single queue to manage the I/O requests. Meanwhile, there is no corresponding optimization strategy for IoT time series databases.

### 2.2. NVM Database

Most of the current database systems are designed for DRAM or disk. Their I/O software stack cannot adapt to the characteristics of NVM devices. HiKV is a hybrid KV store based on DRAM and NVM devices [23], which combines the hash index and B+ tree index. Hash index is used in NVM devices to improve search speed, and the B+ tree index is used in DRAM to support range query. The consistency strategy of two indices was designed to reduce the amount of data written to NVM. BzTree is a latch-free B-tree index structure for NVM devices [24]. It uses the non-volatile property of NVM devices to optimize PMwCAS, which simplifies the complexity of the latch-free index based on single-word CAS, and improves the efficiency of the index. SLM-DB is a single-level merged database [25], which makes full use of the B+ tree and LSM-Tree to improve read and write performance with less write and read amplification. RangeKV is a hybrid KV store based on RocksDB with NVM devices and Flash-based SSD [26]. RangeTab is used to manage L0 level data stored in NVM, increases the L0 capacity, and reduces the level and compression times of LSM-Tree. The hash index of RangeTab is created in advance to reduce the time overhead of accessing the NVM devices, and double buffers are used to reduce the write amplification caused by compression. HiLSM is a hybrid KV store based on NVM devices and Flash-based SSD [27]. The log structure and LSM-Tree are mixed to manage the data. A fine-grained and multi-threaded migration strategy and a filtering strategy were designed to solve write performance crash, performance gap between NVM and Flash-based SSD, and write amplification of LSM-Tree. MatrixKV is a hybrid KV store based on LSM-Tree for DRAM, NVM devices, and Flash-based SSD [28]. A matrix container was designed to migrate the L0 level to NVM devices, which can reduce and accelerate the compression between the L0 and L1 level of LSM-Tree, increase the width of each level, and reduce the write amplification. The cross-row hint search strategy was used to improve read performance. Remix is a KV index to reduce the storage space overhead [29]. It can build a sorted view of KV data spanning multiple table files globally, and it can quickly locate the target key by querying the range of multiple index files without the key comparing and the subsequent key searching. RemixDB is based on Remix [29], which uses compression strategy to significantly improve the write and the range query efficiency of LSM-Tree.

In general, the current research on NVM databases have not covered the NVM device driver and how to adapt it to the characteristics of the IoT series database. Its I/O software stack is still the limitation for the efficiency and there is a redundant or useless overhead of IoT time series data management.

### 2.3. Time Series Database

In order to store and analyze massive time series data efficiently, researchers have developed a type of special time series database to overcome the limitations of the time series data management with general DBMS [30]. LSM-Tree (Log-Structured Merge Tree) is very popular in current time series database management systems [31] such as KairosDB [32], OpenTSDB [33], etc. Meanwhile, InfluxDB [34]’s storage engine TSM-Tree is also an optimization based on LSM-Tree. LSM-Tree is designed for a block storage engine. Its advantage is the great write performance, while its disadvantages are low read performance and more write amplification. It uses an update log to convert random write operations into sequential write and takes full advantage of sequential writing of HDD, but it must reorder written data and compress them layer by layer to reduce the cost of reading. This operation caused more serious write amplification. There are many other improvements in the time series databases. Djamel Edine Yagoubi mentioned a distributed parallel indexing method for time series databases [35], which used the correlation between ordered dimensions and adjacent values to achieve high scalability when having a high performance of similarity query processing. Gorilla is an in-memory distributed time series database [36]. It proposed a three-level in-memory data structure, which is a shared index in memory, which can achieve a high throughput of searching. However, due to the volatility and high cost of memory, it must use HBase to regularly backup and map nodes to ensure its availability in the case of single node or regional failures, and it takes the delta-of-delta and XOR-based compression mechanism to minimize memory space overhead. There are also some time series database management systems based on HDD. To overcome the problems of slow read and write speed of HDD, they designed a series of optimization mechanisms. For instance, LittleTable is a distributed relational database optimized for time series data [2]. To improve write performance, LittleTable spends more than half of its time overhead on seeking data in the tablets to flush data into the HDD at once time together. Therefore, it requires a large buffer. To enhance query efficiency, LittleTable uses two-dimensional clustering of relational tables. One divides the rows by timestamp, so the latest time series data can be retrieved quickly; the other divides the keys by hierarchical structure, so that each partition can be further sorted to realize a fast indexing of time series data with flexible data table model optimization. However, its consistency and durability guarantees are weak and there is no batch delete function because its underlying layer is still a relational database storage engine. ModelarDB is a general-purpose modular distributed time series database management system [37] that stores time series data as a model, hence all operations are optimized on the model’s mode, and multiple models can dynamically adapt to the characteristics of the dataset. ModelarDB relays on Spark and Cassandra to manage time series data to accomplish high-speed writing capabilities, data compression, and scalable online aggregation query processing capabilities. Nevertheless, the model-based design also limits ModelarDB as it can only be used for fixed-frequency time series, and the compression of time series data is lossy compression. Moreover, ModelarDB’s write performance drops sharply when there are many preselected models, as it needs to try all models for each segment of the time series. Peregreen is a distributed modular time series database management system deployed in a cloud environment [38]. It is designed for great-scale historical time series data in the cloud. Peregreen takes a dual storage method that divides the time series data into segments and blocks, then merges them into a three-tier index. It can efficiently achieve queries and calculations with small extra overhead; however, Peregreen also requires a large buffer to provide great write performance, since it bundles the timestamp and value into a pair of compressions. Accordingly, when there is a mixed time series, its compression ability is poor, resulting in the performance of reading data being sharply reduced when using a remote storage device.

However, there is still a lack of optimization for the IoT time series data management, and the current time series database cannot adapt very well to the regular and concurrent writing of massive data with a fixed format. Furthermore, there are some redundant or useless overheads to manage the IoT time series data with them.

## 3. NVM Device Driver Structure for IoT Time Series Databases

**Figure 1:**
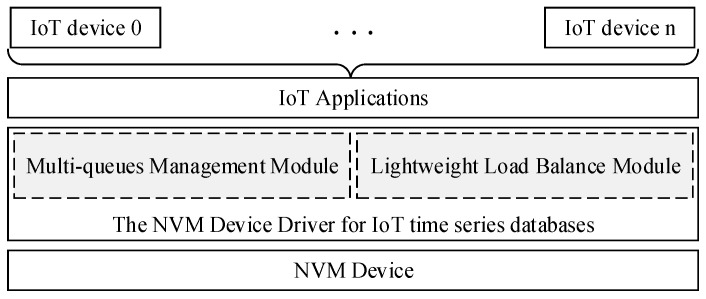
The NVM device driver for IoT time series databases.

There are several concurrent channels in NVM devices to improve the I/O performance [3], and several I/O requests can be processed in parallel by itself. Moreover, the read and write speed of NVM devices is much higher than Flash-based SSD and HDD [6]. However, current storage device drivers generally use serial mode to process I/O requests in turn, which is an important bottleneck for the efficiency of NVM devices. Multicore processors are commonly used to speed up the efficiency of computer systems, but the high I/O performance and throughput of storage systems are also needed to access and store the commands and data of applications. As shown in Figure 1, we present a new architecture of a NVM device driver for IoT time series databases, and there are two main modules in it such as a multi-queue management module and a lightweight load balance module. The multi-queue management module is used to build several I/O queues to handle a large number of I/O requests from the IoT time series database. The lightweight load balance module is used to distribute write requests from different IoT devices reasonably to different I/O processing queues depending on the characteristics of these IoT devices.

The NVM device driver for the IoT time series database uses several queues to manage I/O requests. It can improve the I/O management concurrency of the NVM device driver, and meet the high-speed and concurrent write demand of IoT time series databases. At the same time, some factors such as the data generation frequency of IoT devices have been introduced to optimize the management of I/O queues, which can effectively target the different characteristics of IoT devices and ensure the stable I/O performance of NVM device drivers and reduce the I/O latency of NVM device driver.

## 4. Multi-Queue Management Strategy

There are several concurrent channels in NVM devices, and they can improve the I/O performance through the concurrent reading and writing of multiple channels. However, in the current NVM device driver, the I/O requests received from the generic block layer are still processed serially, which affects the performance of processing IoT time series data in a time series database with NVM devices. Next, there is a big I/O performance gap between the IoT time series database and NVM devices, so it is necessary to improve the concurrency of the NVM device driver. IoT time series data are not allowed to be updated and deleted randomly, so there are fewer read and write conflicts in IoT time series databases compared with general time series databases. Combined with the partition storage strategy for the IoT time series data that we designed in earlier research [8], the read and write conflicts can be effectively avoided in the IoT time series database. This provides favorable conditions for improving the concurrency of NVM device drivers for IoT time series databases. On this basis, we designed a multi-queue management strategy of the NVM device driver for IoT time series databases.

**Figure 2:**
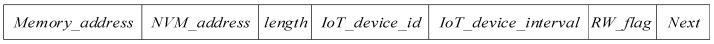
Structure of Tio.

Currently, each block I/O request in the Linux kernel is represented by a Bio [39] structure, which is a common request structure in the Linux kernel and represents an in-flight block I/O operation. With the Bio structure, device drivers can learn the type of operation, related block devices, and other necessary information related to I/O requests. By analyzing the data type and size collected by IoT devices in the introduction part, we changed the data processing unit Bio in the general storage device driver and defined a new structure Tio to manage the IoT time series data in the NVM device driver. As shown in Figure 2, Tio consists of Memory_address, NVM_address, length, etc. Memory_address is the data address in memory, NVM_address is the data address in NVM, length is the bytes to be processed, and IoT_device_id is the identifier of the IoT device corresponding to the I/O request. IoT_device_interval is the time interval of this IoT device for collecting data. RW_flag is used to mark the read or write operations of this I/O request, and Next is the pointer that points to the next I/O request and builds the Tio queue. The interface of the NVM device driver is also modified. The Bio is decomposed to generate several corresponding Tios by its sources, after the NVM device driver receives the Bio submitted from the general block layer in the operating system.

**Figure 3:**
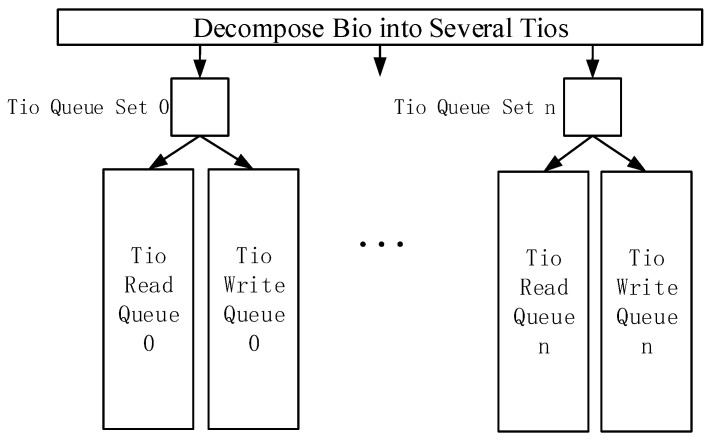
Multi-queue management strategy.

As shown in Figure 3, the I/O processing flow in the NVM device driver is modified to create several sets of Tio queues with multiple threads. Each set of Tio queue contains a write Tio queue and a read Tio queue, and each Tio queue is used to receive and process part of the Tio. Therefore, a single I/O queue in the current storage device driver is converted into multiple sets of Tio queues. In each set of Tio queue, the write Tio queue is prior to the read Tio queue. Each IoT time series data has a timestamp, which enables the NVM device driver to resolve read–write conflicts. The storage space of the NVM device is also divided into several partitions, where each partition is used to store the IoT time series data from different IoT devices.

The multi-queue management strategy can change the processing method of I/O requests in the NVM device driver according to the characteristics of the IoT time series data. Tio is used to adapt the granularity of the IoT time series data instead of Bio with block granularity, which can improve the concurrency of I/O request management. At the same time, several threads are used to build multiple sets of Tio queues with read and write division, and storage space is also divided into several partitions. The concurrency of I/O requests from different IoT devices can be increased. The conflicts of read and write requests can be decreased in NVM device drivers to improve the I/O concurrency of IoT time series databases. In addition, the read and write requests are divided into two queues in each set of Tio queue, and the conflicts of read and write can be decreased in NVM device drivers to improve the I/O concurrency of IoT time series databases.

## 5. Lightweight Load Balance Mechanism Based on Frequency

There are a large number of different types of IoT devices in IoT systems, and their write frequencies to the IoT time series database are also different. There is a serious problem of load balance between different sets of Tio queues. Meanwhile, the write frequency of a single IoT device is fixed, which brings convenience to the load balance of these Tio queue sets in the NVM device driver. In addition, the NVM device has a high read and write speed, which requires a simplification of the NVM device drivers, and minimizes the extra overhead. According to the characteristics of IoT devices, we designed a lightweight load balance mechanism based on frequency.

**Figure 4:**
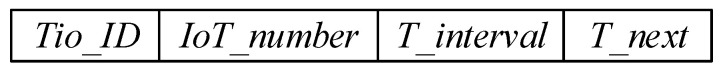
Tio queue set load table.

As shown in Figure 4, we defined the Tio queue set load table Tio_list_load (Tio_ID, IoT_number, T_interval, T_next) to store the current load of each Tio queue set in the NVM device driver. Tio_ID is the identifier of a Tio queue set, and IoT_Number is the number of IoT devices that will send the I/O request to this Tio queue set. T_interval is the sum of the time interval of all IoT devices corresponding to this Tio queue set, and T_next is a pointer that points to the next Tio_list_load node.

The read and write request of each IoT device is managed by a specific Tio queue set. If there is a new IoT device, Equation (1) will be used to calculate the load rate K of all Tio queue sets. The Tio queue set with the highest K will be chosen to accept the I/O request from this new IoT device. The interval of this IoT device will be used to update the T_interval value of the corresponding node in Tio_list_load and the IoT_number will be updated simultaneously.

K = T_interval/IoT_number(1)

The lightweight load balance mechanism based on frequency can dynamically balance the load across Tio queue sets by the characteristics such as the fixed acquisition interval of a single IoT device and different acquisition intervals of different IoT devices. Each IoT device is bound to the corresponding Tio queue set to avoid the periodical collecting load and scheduling, which can reduce the extra overhead for the NVM device driver. The interval characteristic of each IoT device is used to balance the load, which can adapt to the characteristics of the IoT time series database.

## 6. Prototype and Evaluation

### 6.1. Prototype and Test Environment

Intel Optane DC persistent memory is a commercialized NVM device with DIMM interface, and PMEM [27] is the open-source driver for it. We modified the source code of PMEM and added about 800 lines of C code to implement the multi-queue management strategy and lightweight load balance mechanism based on frequency and built the prototype of a new NVM device driver for IoT time series databases named TS-PMEM.

In order to test and analyze the performance of TS-PMEM, two popular NoSQL time series databases named InfluxDB and OpenTSDB, mentioned in the related work section, were installed with PMEM and TS-PMEM for the comparison. Meanwhile, a new IoT time series database for NVM devices from our group named TS-NSM [8] was also used for the test, which can skip the file system layer to shorten the I/O software stack for IoT time series databases. In general, there were six prototypes: InfluxDB + PMEM, OpenTSDB + PMEM, TS-NSM + PMEM, InfluxDB + TS-PMEM, OpenTSDB + TS-PMEM, and TS-NSM + TS-PMEM. Ext4 was used as the file system for InfluxDB and OpenTSDB based on PMEM. The I/O process identifier was used to balance the load across the Tio queue sets for InfluxDB and OpenTSDB based on TS-PMEM, because InfluxDB and OpenTSDB cannot transmit the identifiers of IoT devices to TS-PMEM. All prototypes used Intel Optane DC persistent memory as the storage device to avoid the impact of the storage media.

YCSB-TS is a dedicated tool for testing time series databases. Two typical read–write-mixed workloads named testworkloada and testworkloadb in YCSB-TS were modified by increasing the amount of writing time series data to 10,000 for testing. Each workload consists of load and run phases. During the load phase, testworkloada writes 10,000 time series data with one tag to the prototype and testworkloadb writes 10,000 time series data with three tags. In the run phase, testworkloada performs 1000 random queries and testworkloadb performs 1000 random time-range queries. In addition, there are 250 scans, counts, sums, and average calculations on the query results in the run phase of testworkloadb. Each item was tested 10 times to calculate the average value as the result, and the server was restarted and the cache was disabled before each test to eliminate the effect of the cache on the test results. The number of Tio queue sets was set to 4 in prototype.

One server was used to test the six prototypes, and the hardware and software configurations of this server are shown in Table 1. In order to simulate the concurrency of I/O requests from IoT devices to the IoT time series database and to avoid the effects of device connectivity, data loss, network, and other uncertainties in the IoT system, we used multiple terminals in the server described below to simulate multiple IoT time series data sources, each of which can appropriately simulate the time series data flow of a typical IoT system. We installed the load used for testing in each terminal, and then unified the operations of each terminal to initiate requests for reading and writing time series data at the same time. When the IoT time series data source is 1, it means that the IoT data flow and that of the system is small and the system load is at a low level, and we set up four threads in YCSB-TS to test in this case. As the number of IoT time series data source increases, the load of the system also increases. Similarly, when the IoT time series data source is 6, it means that the system is under high load and needs to process a large number of requests for IoT time series data, so we set 24 threads for YCSB-TS to test.

### 6.2. Write Throughput

**Figure 5:**
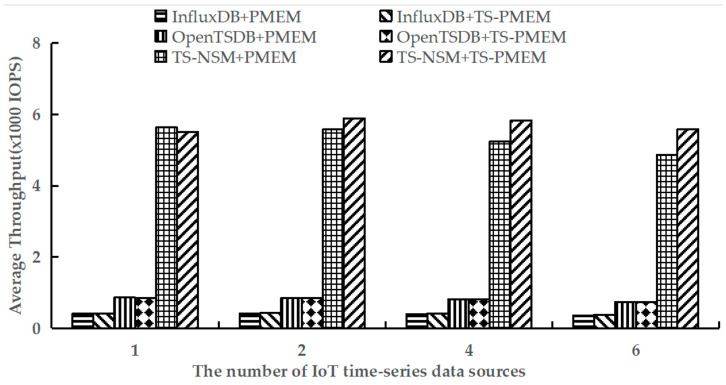
Average write throughput under testworkloada.

**Figure 6:**
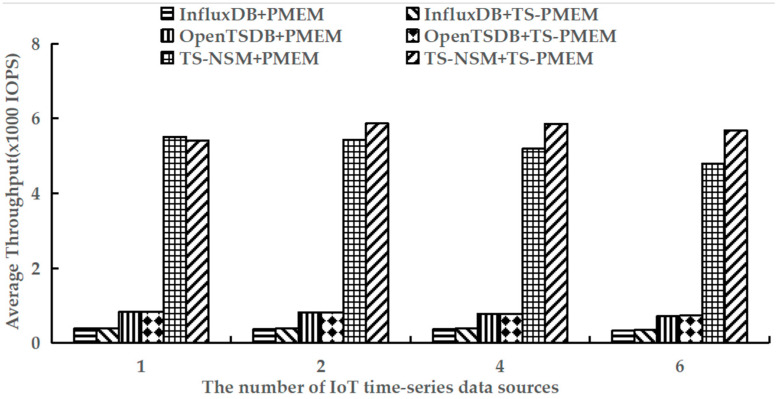
Average write throughput under testworkloadb.

Because all write requests are in the load phase, the load phase of testworkloada and testworkloadb in YCSB-TS was performed to test the average write throughput of TS-NSM + PMEM, InfluxDB + PMEM, OpenTSDB + PMEM, TS-NSM + TS-PMEM, InfluxDB + TS-PMEM, and OpenTSDB + TS-PMEM, respectively. The test results are shown in Figure 5 and Figure 6.

Figure 5 shows the results of executing the load phase using testworkloada. It can be found that TS-PMEM can effectively improve the write throughput of TS-NSM, InfluxDB, and OpenTSDB compared with PMEM when there are multiple IoT time series data sources. The increase was 5.5–14.8%, 2.8–5.6%, and 0.4–0.6%, respectively. This indicates that TS-PMEM can adapt to the characteristics of multiple IoT devices to write data concurrently and improve the throughput of IoT time series databases. TS-NSM provides the highest write throughput because TS-NSM is a new time series database designed for IoT systems and NVM devices and can shorten the I/O software stack. Meanwhile, its write throughput improvement was also the highest. This also shows that the concurrency and low extra software overhead are both effective ways to improve the efficiency of applications based on NVM devices. The write throughput improvement of OpenTSDB was the lowest because it caches a small size of write requests and composes them into large blocks for writing. This strategy can reduce I/O congestion and latency of NVM device drivers, but it affects the function of the multi-queue mechanism in TS-PMEM. Its write throughput was higher than InfluxDB, but still far worse than TS-NSM. However, when there was only one IoT time series data source, the write throughput of TS-NSM, InfluxDB, and OpenTSDB based on TS-PMEM were all lower than the prototypes based on PMEM. This is because there is an additional time overhead of the multi-queue management strategy and the lightweight load balance mechanism based on frequency in TS-PMEM. However, the write throughput decrease was small, ranging from 0.3% to 2%. When the amount of IoT time series data sources increased from 1 to 2, the TS-PMEM could improve the write throughput of TS-NSM and InfluxDB. In contrast, PMEM decreased the write throughput of TS-NSM and InfluxDB. In addition, write throughput decreased across all prototypes based on PMEM with the increase in IoT time series data sources, but the magnitude of write throughput decreases can be reduced for the prototype based on TS-PMEM. These results indicate that TS-PMEM can effectively improve concurrent writing throughput of IoT time series databases.

Figure 6 is the result of executing the load phase using testworkloadb. Similar to using testworkloada, TS-PMEM improved the writing throughput of all prototypes when there were multiple IoT time series data sources. Compared with testworkloada, more data is written to the prototype at one time using testworkloadb, which results in a slight decrease in write throughput for all prototype systems. However, the ratio in the improvement in write throughput by TS-PMEM was higher than using testworkloada. It improved write throughput by 8.0–18.6% for InfluxDB and 3.1–7.8% for TS-NSM, which was higher than when using testworkloada. Moreover, when there are more IoT time series data sources in prototypes, the more I/O conflicts result in the write throughput decrease of TS-NSM, InfluxDB and OpenTSDB based on PMEM. When the amount of IoT time series data sources increases from 1 to 6, their throughput decreases by 13%, 13.5% and 12.7%, respectively. This indicates that there is the concurrency limitation of current NVM device drivers for IoT time series databases. With TS-PMEM, the write throughput of TS-NSM and InfluxDB increased first and then decreased. When the number of IoT time series data sources increased from 1 to 6, the write throughput of TS-NSM, InfluxDB, and OpenTSDB based on TS-PMEM decreased by only −5%, 6%, and 11.6%, respectively. Its decrease was smaller than the corresponding prototypes based on PMEM. These results further indicate that TS-PMEM has higher concurrency and can better adapt to the IoT time series database.

### 6.3. Write Latency

**Figure 7:**
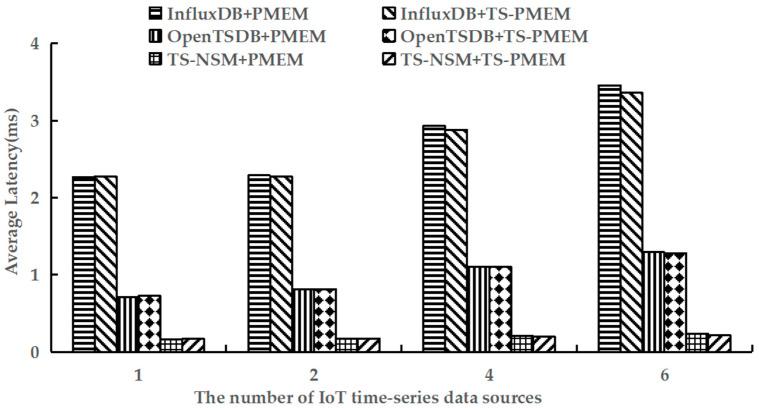
Average write latency under testworkloada.

We used YCSB-TS to perform the load phase of testworkloada and test the average write latency of six prototype systems. The results are shown in Figure 7.

As shown in Figure 7, TS-PMEM can significantly reduce the write latency of time series data when there are multiple IoT time series data sources. Compared with PMEM, the write latency of TS-NSM, InfluxDB, and OpenTSDB based on TS-PMEM was reduced by 1.7–8.3%, 0.5–2.8%, and 0.2–0.9%, respectively. This verifies that TS-PMEM can effectively reduce the write latency in IoT time series databases. As the number of IoT time series data sources increased, the write latency of all prototypes increased to varying degrees. When the number of IoT time series data sources was 6, the write latency of TS-NSM, InfluxDB, and OpenTSDB with PMEM was 44.6%, 52.5%, and 81.0% higher than those with only one IoT time series data source, respectively. However, the write latency of TS-NSM, InfluxDB, and OpenTSDB based on TS-PMEM only increased 30.2%, 47.8%, and 76.3%, respectively. This fully verifies that TS-PMEM has a more stable write performance than PMEM and can accommodate concurrent writing in an IoT time series database.

### 6.4. Query Throughput

**Figure 8:**
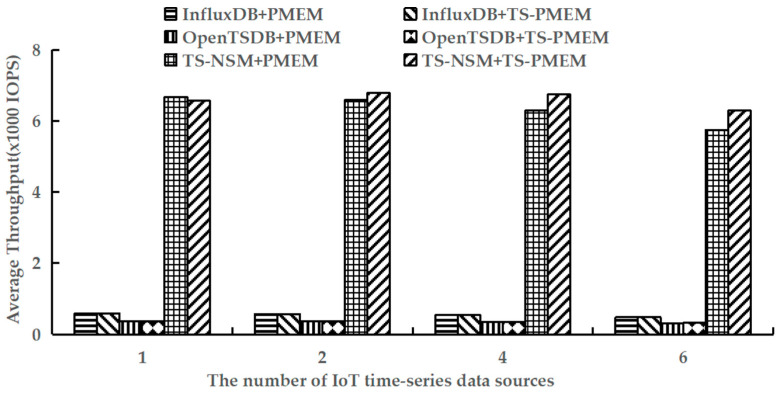
Average random query throughput under testworkloada.

**Figure 9:**
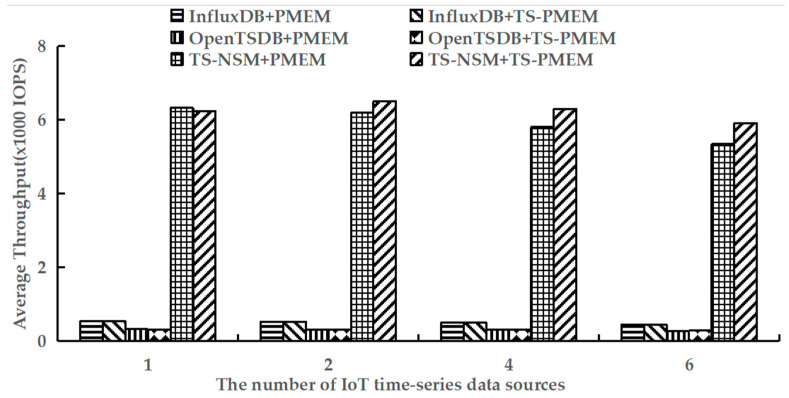
Average range query throughput under testworkloadb.

Using YCSB-TS to perform the run phases of testworkloada and testworkloadb to test the average query throughput of six prototypes. These workloads contain read and write operations. The results are shown in Figure 8 and Figure 9.

Figure 8 shows the results of the average throughput when executing random queries in six prototypes. It can be seen that TS-PMEM can effectively improve the random query throughput of TS-NSM and OpenTSDB when there are multiple IoT time series data sources, which were 3.0–9.5% and 0.4–1.8% higher than TS-NSM and OpenTSDB based on PMEM, respectively. InfluxDB’s random query throughput only improved by 0.9% because InfluxDB caches data to improve the efficiency of subsequent queries and reduce I/O requests to NVM device drivers. Similar to the result of write throughput, as the number of IoT time series data sources increased the random query throughput of TS-NSM, InfluxDB, and OpenTSDB based on PMEM decreased continuously. When the number of IoT time series data sources increased from 1 to 6, their random query throughput decreased by 14.1%, 17.8%, and 14.2%, respectively. In contrast, the random query throughput of TS-NSM, InfluxDB, and OpenTSDB based on TS-PMEM increased first and then slowly decreased. When the number of IoT time series data sources increased from 1 to 6, their random throughput decreased by only 4.4%, 17.6%, and 11.6%, respectively. These results indicate that TS-PMEM has a better ability to adapt to concurrent queries in IoT time series database and higher query throughput than PMEM.

Figure 9 shows the average throughput of range query with testworkloadb. Similar to random query results, TS-PMEM could improve the range query throughput of TS-NSM and OpenTSDB when there are multiple IoT time series data sources, while InfluxDB still had a small increase in range query throughput due to the read cache. However, unlike random query results, the range query throughput improvement in TS-NSM and OpenTSDB based on TS-PMEM was higher than those based on TS-PMEM, reaching 5.0–10.6% and 0.6–1.4%, respectively. This is because the range query needs to read larger amounts of data, which can increase the efficiency of TS-PMEM. These results indicate that TS-PMEM can improve the efficiency of range query and adapt to the characteristics of the IoT time series database.

### 6.5. Query Latency

**Figure 10:**
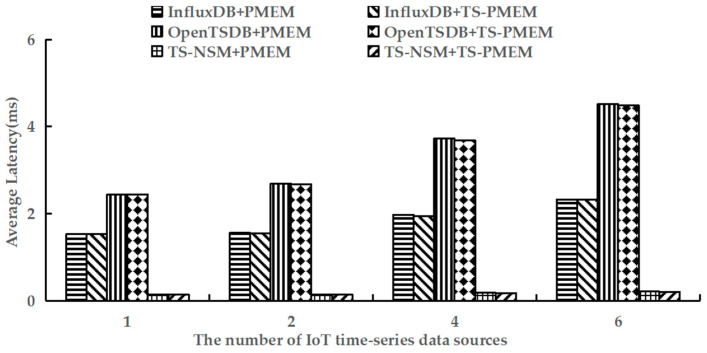
Average random query latency under testworkloada.

Finally, YCSB-TS was used to perform the run phase of testworkloada and test the average query latency of six prototypes. The test results are shown in Figure 10.

The results in Figure 10 show that TS-PMEM could reduce the average latency of random queries for TS-NSM, InfluxDB, and OpenTSDB by 2.0–6.4%, 0.5–1.4%, and 0.7–0.9%, respectively, when there are several IoT time series data sources. The average query latency of the six prototype systems increased as the number of IoT time series data sources increased. The increase of random query latency of prototypes based on TS-PMEM was smaller than others based on PMEM. These results demonstrate that TS-PMEM can effectively reduce the latency of concurrent random queries in IoT time series databases and improve the response speed of IoT applications.

## 7. Conclusions and Future Works

We analyzed the characteristics of IoT time series databases and NVM devices as well as the bottlenecks of existing NVM drivers in IoT time series data storage. A new NVM device driver was designed for IoT time series databases. The multi-queue management strategy and the lightweight load balance mechanism based on frequency were used to shorten the I/O software stack and improve the concurrency of NVM devices. Based on the open-source NVM device driver, a prototype of the NVM device driver for IoT time series databases was implemented named TS-PMEM. Six prototypes were built to evaluate through the YCSB-TS to verify the efficiency of the TS-PMEM. The experimental results show that TS-PMEM can effectively improve the read and write throughput of IoT time series data and reduce the processing latency compared with PMEM.

In the current design, the efficiency is the main object for load balance. How to balance the load between CPU and NVM devices will be studied in the future.

## Figures and Tables

**Table 1:** Configuration of evaluation.

Components	Configuration
CPU	Intel Xeon Platinum 8260 M 2.30 G
Memory	128 GB
NVM device	2 × 128 GB Intel Optane DC Persistent Memory
Disk	256 GB NVMe SSD
OS	CentOS 7.0, Kernel 4.4.112
